# Improving the EMA Binding Test by Using Commercially Available Fluorescent Beads

**DOI:** 10.3389/fphys.2020.569289

**Published:** 2020-09-15

**Authors:** Andreas Glenthøj, Alaa Sharfo, Christian Brieghel, Amina Nardo-Marino, Henrik Birgens, Jesper Brix Petersen

**Affiliations:** ^1^Danish Center for Hemoglobinopathies, Department of Hematology, Copenhagen University Hospital, Herlev and Gentofte Hospital, Herlev, Denmark; ^2^Department of Hematology, Rigshospitalet, Copenhagen, Denmark

**Keywords:** hereditary spherocytosis, EMA binding test, hemolysis, anemia, membranopathy, ektacytometry

## Abstract

Hereditary spherocytosis (HS) is a common anemia caused by germline mutations in red blood cell cytoskeleton proteins. The flow cytometry-based eosin-5′-maleimide (EMA) binding test is most frequently employed for reliable diagnostics. To perform this test, a number of healthy and ideally also age-matched controls are required, which can be challenging and complicates interlaboratory comparisons. To overcome this limitation, we modified the EMA binding test by replacing healthy controls with commercially available fluorescent beads. Blood samples from 289 individuals with suspected HS were analyzed using the EMA binding test with fluorescent beads and benchmarked against regular EMA binding test using two control samples. Using osmotic gradient ektacytometry as validation, 112 individuals (38.8%) were diagnosed with HS. Performance of the modified EMA binding test was not compromised (accuracy 90.3%) compared to EMA binding test using matched controls (accuracy 88.6%). Based on these findings, we conclude that the modified EMA binding test with fluorescent beads is an attractive alternative, especially in laboratories without easy access to matched controls. Furthermore, as fluorescent beads are stable and easily commutable, they could facilitate both interlaboratory comparisons and quality assessment programs.

## Introduction

Hereditary spherocytosis (HS) is one of the most frequent hereditary hemolytic disorders in Caucasians, affecting approximately 1:2000. Typically, affected individuals present with direct antiglobulin test (DAT) negative hemolytic anemia, increased mean corpuscular hemoglobin concentration (MCHC), and palpable splenomegaly, as well as a known family history of hemolytic anemia and gallbladder disease. In severe cases, transfusions are required from early childhood. Splenectomy is the main therapeutic option for symptomatic patients (Iolascon et al., 2017).

For decades, the laboratory diagnosis of HS was based on the manual osmotic fragility (OF) test. This obsolete test measures the degree of hemolysis after exposing red blood cells (RBCs) to a salt solution of diminishing tonicity, a procedure that is laborious. Sensitivity and specificity of the OF test are limited, and it has been estimated that approximately 20% of mild cases of HS remain undiagnosed when using only OF test (Perrotta et al., 2008). Most laboratories have now replaced the OF test with new and more technically demanding methods such as the flow cytometry-based eosin-5′-maleimide (EMA) binding test and osmotic gradient ektacytometry.

Osmotic gradient ektacytometry is the gold standard for measuring RBC deformability. In most laboratories, however, ektacytometry has not been accessible until recently. A new generation of ektacytometers is now available, enabling this diagnostic principle to be applied on a broader scale (Da Costa et al., 2016; Llaudet-Planas et al., 2018). Osmotic gradient ektacytometry relies on laser diffraction analysis and allows for a direct and accurate measurement of alterations in the deformability of RBCs under a predetermined shear stress. Unfortunately, the test does not distinguish between HS and other spherocytic conditions such as autoimmune hemolytic anemia (Lazarova et al., 2017) and a DAT is needed to differentiate these.

In most cases, HS is caused by germline mutations in genes encoding RBC cytoskeleton proteins such as α-spectrin, β-spectrin, band 3, ankyrin, and protein 4.2. Even so, upfront genetic diagnostics rarely assists diagnostic workup and is only employed on a case-by-case basis (King et al., 2015). This is due to low sensitivity, difficulties with interpretation of identified – often private -variants, and high costs. As a result, functional testing of RBCs remains the gold standard.

Most laboratories rely on the EMA binding test as the primary screening test for HS. This test provides excellent sensitivity and specificity of >86% and often even >95% (King et al., 2000; Bianchi et al., 2012; Park et al., 2014; Joshi et al., 2016; Arora et al., 2018). The principle of the EMA binding test is measurement of RBC fluorescence intensity after incubation with EMA; a fluorescent substance that binds to RBC membrane-associated proteins, mainly band 3 (King et al., 2004). In individuals with HS, the reduced membrane surface area of the RBCs, and thereby the amount of band 3, is typically reduced (Perrotta et al., 2008). Thus, RBCs from individuals with HS can be distinguished from normal RBCs due to lower mean fluorescence intensity (MFI). The EMA binding test is relatively simple to perform, and results are available within a few hours. Generally, the MFI of the RBCs in individuals with suspected HS is compared to that of normal controls, typically three to six healthy individuals (Hunt et al., 2015). Ideally, but not mandatory, blood from healthy controls should be age-matched and drawn at the same time as the diagnostic blood sample (Falay et al., 2018). Unfortunately, this approach is often not feasible as many laboratories rely on control samples from blood donors, hospital personal, and even leftover blood samples from patients considered hematologically healthy. Especially in children, finding normal age-matched controls can be a challenge. The use of variable and non-commutable reference materials greatly complicate definition of reference ranges, interlaboratory comparisons, and quality assessment programs (Miller et al., 2011; King et al., 2015).

In this study, we investigate the performance and robustness of a modified EMA binding test by replacing fresh blood samples from healthy controls with commercially available fluorescent beads, in order to simplify HS diagnostics.

## Methods

### Samples

We included blood samples from all patients referred to our laboratory with suspected HS between February 6, 2017 and September 4, 2019. The Danish Center for Hemoglobinopathies covers >75% of the Danish population and performs the vast majority of membranopathy diagnostics in the country. Travel control was in general not included due to local practices. Samples older than 48 h were discarded.

To derive a calibration factor (CF), we further obtained blood samples from 71 healthy participants in the Copenhagen General Population Study (Warny et al., 2020).

All blood samples were analyzed by osmotic gradient ektacytometry and EMA binding test as described below. Samples were transported and stored cold.

DAT was performed locally. As osmotic gradient ektacytometry is not able to distinguish between immune hemolysis and HS (Da Costa et al., 2016), we considered patients with a positive DAT to have immune hemolysis. Clinical information was only scarcely available.

### Eosin-5′-Maleimide Binding Test

The EMA binding test was performed on EDTA-stabilized blood within 48 h of sampling. All EMA binding tests were performed on a FACS Canto II (BD Biosciences, Franklin Lakes, NJ, United States) using standard filter options (530/30).

The labeling of RBCs with EMA and flow cytometry was performed as previously described by others (King et al., 2000, 2004). Albeit, instead of using six healthy age-matched controls, we compared samples to commercially available fluorescent beads.

In detail, each sample was included in an EMA-experiment which, in addition to two control samples, also included two types of commercially available fluorescent beads. Firstly, one drop of FluoroSpheres K0110 beads (calibration beads; Agilent Technologies Inc., Palo Alto, CA, United States) diluted in 500 μL phosphate-buffered saline solution (PBS) was used in every experiment to calibrate the photo multiplier setting of the flow cytometer to a constant value. This was done to ensure that the fluorescence level from all experiments was comparable. Secondly, each sample was compared to one drop of mid-range FL1 Rainbow Fluorescent Particle beads (Rainbow beads; BD Biosciences) diluted in 500 μL PBS.

Eosin-5′-maleimide for the individual patient was calculated as:

$${\text{EMA}}_{\text{beads}}\left(\%\right)=\left(1-\frac{{\text{MFI}}_{\text{patient}}}{{\text{MFI}}_{\text{rainbow}}\times \mathrm{CF}}\right)\times 100\%$$

Rainbow bead MFI was slightly higher than the average fluorescence level of controls. Therefore, a constant CF was calculated based on the average of control samples from 71 healthy controls:

$$\text{CF}=\text{Mean}\,\left(\frac{{\text{MFI}}_{\text{healthycontrols}}}{{\text{MFI}}_{\text{rainbow}}}\right)$$

The reason for this CF is solely to ensure that the EMA value obtained can be compared with the EMA values stated in the literature. New lots of rainbow beads should be calibrated toward the previous to ensure consistency of the EMA values calculated. Otherwise, a new CF should be calculated.

Two healthy controls were routinely included with each sample as an extra safety measure and a more traditional EMA value was calculated using these parameters:

$${\text{EMA}}_{\text{controls}}\left(\%\right)=\left(1-\frac{{\text{MFI}}_{\text{patient}}}{\mathrm{Mean}\,\left({\mathrm{MFI}}_{\mathrm{control1}}+{\text{MFI}}_{\mathrm{control2}}\right)}\right)\times 100\%$$

Consequently, the value of EMA_controls_ was calculated using only two controls contrary to the six usually employed.

A detailed step-by-step protocol is freely available online at https://www.protocols.io/view/eosin-5-maleimide-ema-binding-test-with-fluorecent-bigdkbs6 (Glenthøj and Petersen, 2020).

### Osmotic Gradient Ektacytometry

Osmotic gradient ektacytometry was performed within 48 h of sampling. All analyses were performed on a LoRRca ektacytometer (RR Mechatronics, Zwaag, Netherlands) as previously described (Da Costa et al., 2016).

### Statistical Analyses

We used linear regression with Pearson correlation to evaluate linear modeling. We used Fisher’s exact test to compare baseline characteristics of categorical variables and Wilcoxon signed rank test to compare numerical variables. Statistical analyses were performed in ‘R’ version 4.0.0 (R Core Team, 2017) using the ggplot2 package for charts, tableone baseline characteristics, pROC (Robin et al., 2011) and plotROC packages for receiver operating characteristic (ROC) analyses, and caret package for accuracy calculations.

## Results

### EMA With Fluorescent Beads in Healthy Controls

**FIGURE 1:**
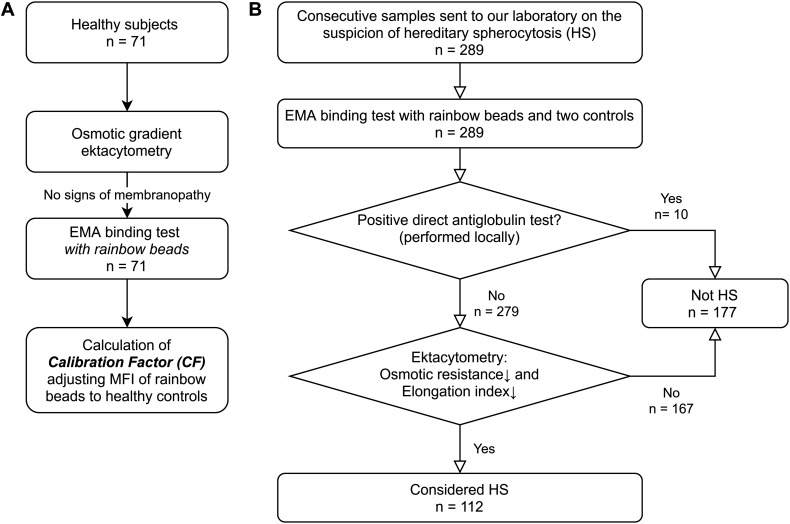
Study flowchart **(A)** Healthy subjects participating in the Copenhagen General Population Study were subjected to osmotic gradient ektacytometry and EMA binding test with rainbow beads. These subjects were only used to calculate a constant Calibration Factor (CF) normalizing mean fluorescent intensity (MFI) of rainbow beads to the mean of healthy controls. **(B)** 289 patients had samples sent to our laboratory on suspicion of hereditary spherocytosis (HS). EMA binding test and osmotic gradient ektacytometry was performed. Patients without a positive direct antiglobulin test and spherocytosis on osmotic gradient ektacytometry were labeled as HS to test the accuracy of the EMA test.

Characteristics of the 71 control subjects from the Copenhagen General Population Study are shown in Table 1. Blood samples from healthy controls were subjected to EMA binding test with rainbow beads and osmotic gradient ektacytometry (Figure 1A). Neither EMA (MFI reduction) nor osmotic gradient ektacytometry [decreased osmotic resistance and elongation index (Llaudet-Planas et al., 2018)] indicated a membranopathy in any of the 71 subjects, when compared to the other 70 subjects (data not shown). Mean MFI_controls_ was 12,068 ± 599 (SD) and mean MFI_rainbow_ was 14,900 ± 96 (SD). CF was thus 0.81 (12,068/14,900).

**TABLE 1:** Characteristics of healthy controls from the Copenhagen General Population Study used for calculating a calibration factor as well as 289 patients with samples sent to our laboratory for diagnostics of hereditary spherocytosis (HS).

	**Control**	**HS**	**Not HS**	***P***
N	71	112	177	
Sex = Male (%)	36 (50.7)	59 (52.7)	71 (40.1)	0.078
resistance and elongation	67.80 [56.35, 75.35]	19.85 [1.70, 43.92]	37.70 [13.30, 62.00]	<0.001
DAT available, n (%)	0 (0.0)	48 (42.9)	139 (78.5)	<0.001
EI_min_, mean (SD)	0.15 (0.04)	0.13 (0.03)	0.14 (0.04)	0.007
O_min_, mean (SD)	148.99 (5.68)	177.33 (13.04)	158.19 (14.30)	<0.001
EI_max_, mean (SD)	0.61 (0.01)	0.55 (0.03)	0.60 (0.02)	<0.001
O_max_, mean (SD)	299.08 (13.69)	339.04 (23.98)	321.85 (23.64)	<0.001
EI_hyper_, mean (SD)	0.30 (0.00)	0.28 (0.02)	0.30 (0.01)	<0.001
O_hyper_, mean (SD)	455.48 (13.05)	455.50 (26.21)	468.43 (23.59)	<0.001
Area, mean (SD)	167.66 (5.33)	139.80 (14.02)	162.11 (10.04)	<0.001

*Age is shown as median with interquartile range (IQR). The two controls per patient are not shown. Sex, age, and DAT were compared using Kruskal–Wallis Rank Sum Test. Ektacytometry parameters (EI_min_, O_min_, EI_max_, O_max_, EI_hyper_, O_hyper_, and Area) were compared using *t*-test.*

### EMA With Fluorescent Beads for Diagnostics of HS

We included blood samples from 289 individuals with suspected HS. In accordance with our inability to find age-matched controls, individuals with suspected HS were significantly younger than the healthy controls (Table 1). DAT was available in 187 of the 289 patients (64.7%). Based on osmotic gradient ektacytometry [decreased osmotic resistance, decreased elongation index and no positive DAT (Llaudet-Planas et al., 2018)], 112 individuals were diagnosed with HS (Figure 1B). Patient characteristics are summarized in Table 1. Patients with HS were significantly younger with fewer available DAT as compared to individuals without HS.

Linearity of the fluorescence was evaluated by changing the photo multiplier of the flow cytometer settings to alter the MFI_patient_ from the standard value to 0.1 and 10 times this value. The difference in MFI_patient_/MFI_rainbow_ ratio was negligible over this range, indicating that minute adjustments of photo multiplier may not be necessary (data not shown).

**FIGURE 2:**
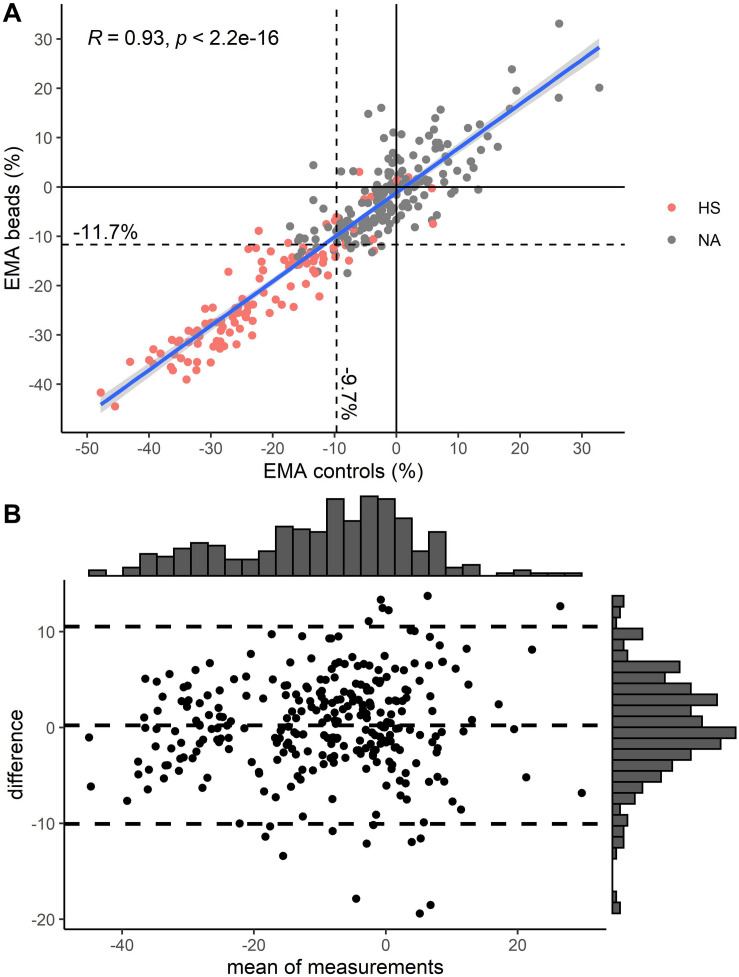
**(A)** Comparison of EMA using two controls vs. EMA using rainbow beads. Pearson correlation (R) is depicted in upper left corner. Color indicates diagnosis of hereditary spherocytosis (HS) by osmotic gradient ektacytometry (Figure 1B). **(B)** Modified Bland-Altman plot for EMA using two controls vs. EMA using rainbow beads. Unit on both axes is percent mean fluorescence intensity compared to healthy controls. Striped lines indicate mean and 1.96 standard deviations above and below that. Histograms are depicted on top and right axes.

A comparison between EMA_beads_ and traditional EMA_controls_ showed excellent correlation between the two methods [Figure 2; Pearson correlation coefficient = 0.93 (95% CI: 0.91–0.94)].

**FIGURE 3:**
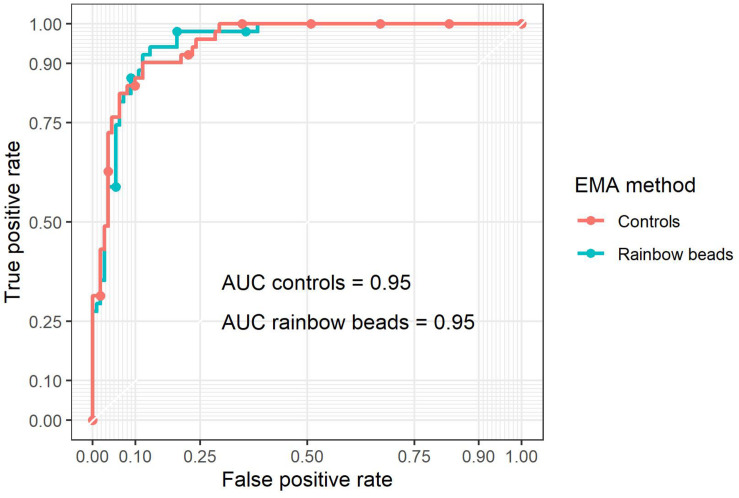
Receiver operating characteristic (ROC) curves of EMA binding test by either two controls (red) or rainbow beads (blue) using osmotic gradient ektacytometry as the gold standard (Figure 1B).

Receiver operating characteristic analysis was performed for EMA_beads_ and EMA_controls_ (Figure 3) with an optimal cut-off value of −11.6 and −9.7%, respectively (Figure 2A). Rainbow beads showed slightly better specificity, positive predictive value, and accuracy compared to using two healthy controls (Tables 2, 3).

**TABLE 2:** Confusion matrix for EMA binding test with either **(A)** rainbow beads or **(B)** two controls.

**(A)**				
		**Ektacytometry**		
**EMA rainbow**		**HS**	**Not HS**	
	HS	97	13	110
	Not HS	15	164	179
		112	177	

**(B)**				
		**Ektacytometry**		
**EMA controls**		**HS**	**Not HS**	

	HS	99	20	119
	Not HS	13	157	170
		112	177	
				

**TABLE 3:** Sensitivity, specificity, positive predictive value (PPV), negative predictive value (NPV), and accuracy for EMA binding test with either rainbow beads or two controls.

	**Sensitivity**	**Specificity**	**PPV**	**NPV**	**Accuracy**
EMA rainbow	0.866	0.927	0.882	0.916	0.903
EMA controls	0.884	0.887	0.832	0.924	0.886

The MFI of the two controls differed by more than 1000 in 87 patients which may have caused low performance when calculating EMA_controls_. After excluding these patients, we found no substantial change in the correlation between EMA_beads_ and EMA_controls_ [Supplementary Figure S1; Pearson correlation coefficient = 0.95 (95% CI: 0.93–0.98)], the ROC analysis (Supplementary Figure S2), or the accuracy of the EMA_controls_ test (Supplementary Tables S1, S2).

## Discussion

In this study, we assessed whether commercially fluorescent beads can replace the need for numerous control samples in the EMA binding test with the aim of simplifying HS diagnostics. Our laboratory, and likely many other laboratories, struggles to find suitable control samples for the EMA test and even with age-matched controls, variation is often considerable. Fluorescent beads are easily accessible and could facilitate interlaboratory comparisons as well as quality assessment programs without the potential errors associated with the use of controls.

Using fluorescent beads, we demonstrate that EMA binding test without readily available healthy controls is feasible and non-inferior to a traditional approach (Figure 2 and Tables 2, 3). We choose to include a CF, which translate the EMA ratio between the MFI of the patient and the rainbow beads into values familiar from the traditional EMA binding test. Theoretically, this is not necessary and the uncorrected MFI ratio between a patient and the rainbow beads could just as well be used for diagnostics.

Due to our decade long standard practice of fluorescent bead EMA, we only use two healthy controls as an extra safety control. As three to six controls are normally employed, this may have weakened the performance of the traditional EMA test in this study (Hunt et al., 2015). We tried to minimize this weakness in our study by removing patients, where the controls differed considerably. This did not substantially alter our results (Supplementary Figures S1, S2 and Table S1). Additionally, clinical information was generally not available to assist the diagnosis of HS. Thus, for the purpose of this study, the reference diagnosis of HS was based on osmotic gradient ektacytometry supplemented by locally performed DAT (Llaudet-Planas et al., 2018). Differential diagnoses include immune hemolysis avoiding detection by a traditional DAT and non-HS membranopathies such as congenital dyserythropoietic anemia type II. Nonetheless, the purpose of our study was not to cement the already established diagnostic sensitivity and specificity of the EMA binding test (Bianchi et al., 2012), but to demonstrate a non-inferior approach to the EMA test in which healthy controls are replaced by commercially available fluorescent beads.

While replacing control samples with fluorescent beads is an attractive alternative, this approach requires regular calibration to ensure that the ratio between the beads and healthy controls does not change. We routinely perform such reviews after changing batches of the fluorescent beads or EMA. Fluorescence of the EMA dye should be stable up to 6 months at −80°C (Mehra et al., 2015). In our diagnostic setting, acquiring healthy controls for quality checks scheduled months apart is more easily implemented than retrieving suitable controls for each EMA binding test. This is particularly difficult with young patients, and we suspect that many other laboratories have similar difficulties obtaining age-matched control blood from healthy children.

In conclusion, our results indicate that commercially available fluorescent beads can eliminate the need for samples from healthy controls in the EMA binding test without compromising test performance. These results require external validation before employing into clinical practice.

## Data Availability Statement

The raw data supporting the conclusions of this article will be made available by the authors, without undue reservation.

## Ethics Statement

Use of blood from healthy controls from the Copenhagen General Population Study was used in accordance with approval from the Danish Ethical Committee (H-KF-01–144/01). Written informed consent was obtained from all participants. All patients or their parents consented to diagnostic tests for hemolytic anemia including tests for hereditary spherocytosis. Data was stored and handled in according with permission from the Danish Data Protection Agency (10122009 HEH-L.HB).

## Author Contributions

HB, AN-M, JP, and AG planned this study. JP performed EMA and ektacytometry analyses. AS, CB, JP, and AG performed the statistical analyses. AG and JP analyzed data and wrote the manuscript. AG prepared the figures. All authors contributed to the final approved version of this report.

## Conflict of Interest

The authors declare that the research was conducted in the absence of any commercial or financial relationships that could be construed as a potential conflict of interest.

## Supplementary Material

FIGURE S1 87 of the 289 patients had controls with mean fluorescence intensity difference >1000 and were discarded. **(A)** Comparison of EMA using two controls vs. EMA using rainbow beads. Pearson correlation (R) is depicted in upper left corner. Color indicates diagnosis of hereditary spherocytosis (HS) by osmotic gradient ektacytometry (Figure 1B). **(B)** Modified Bland-Altman plot for EMA using two controls vs. EMA using rainbow beads. Unit on both axes is percent mean fluorescence intensity compared to healthy controls. Striped lines indicate mean and 1.96 standard deviations above and below that. Histograms are depicted on top and right axes.

FIGURE S2 Receiver operating characteristic (ROC) curves of EMA binding test either two controls or rainbow beads using osmotic gradient ektacytometry as gold standard (Figure 1B). 87 of the 289 patients had controls with mean fluorescence intensity difference >1000 and were discarded.

TABLE S1 Confusion matrix for EMA binding test with either **(A)** rainbow beads or **(B)** two controls. Contrary to Table 2, patients where the controls differed the most in mean fluorescence intensity difference (>1000; 87 of the 289 patients) were discarded.

TABLE S2 Sensitivity, specificity, positive predictive value (PPV), negative predictive value (NPV), and accuracy for EMA test with either rainbow beads or two controls for EMA binding test with either rainbow beads or two controls in patients. Contrary to Table 3, patients where the controls differed the most in mean fluorescence intensity difference (>1000; 87 of the 289 patients) were discarded.
