# Supplementary figures and images for: Improving the EMA Binding Test by Using Commercially Available Fluorescent Beads
Source: Front Physiol. 2020 Sep 15;11:569289. doi: 10.3389/fphys.2020.569289 (PMC7522531; doi:10.3389/fphys.2020.569289)

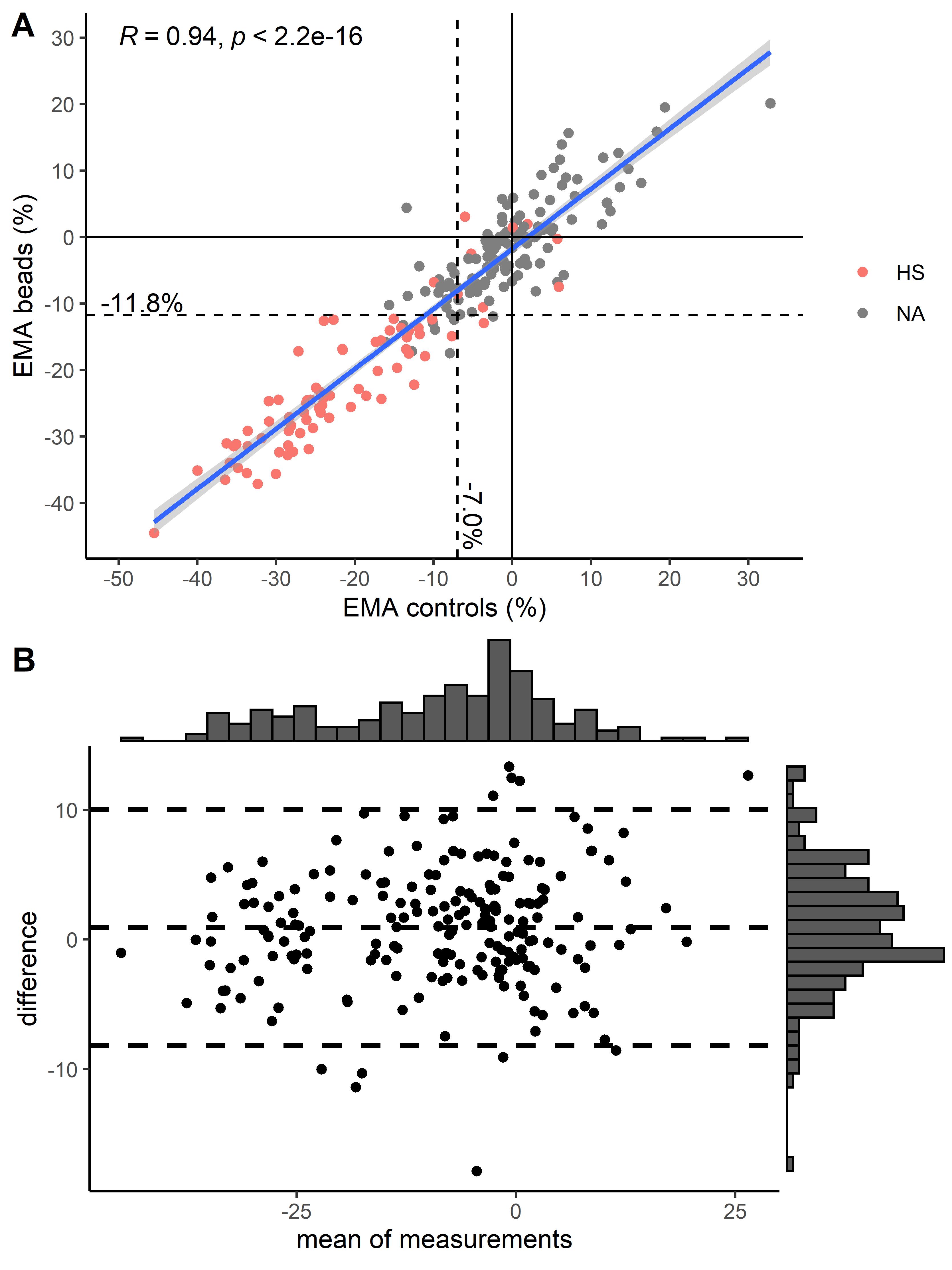

Supplement: FIGURE S1 — 87 of the 289 patients had controls with mean fluorescence intensity difference >1000 and were discarded. (A) Comparison of EMA using two controls vs. EMA using rainbow beads. Pearson correlation (R) is depicted in upper left corner. Color indicates diagnosis of hereditary spherocytosis (HS) by osmotic gradient ektacytometry (Figure 1B). (B) Modified Bland-Altman plot for EMA using two controls vs. EMA using rainbow beads. Unit on both axes is percent mean fluorescence intensity compared to healthy controls. Striped lines indicate mean and 1.96 standard deviations above and below that. Histograms are depicted on top and right axes. [file Image_1.JPEG]

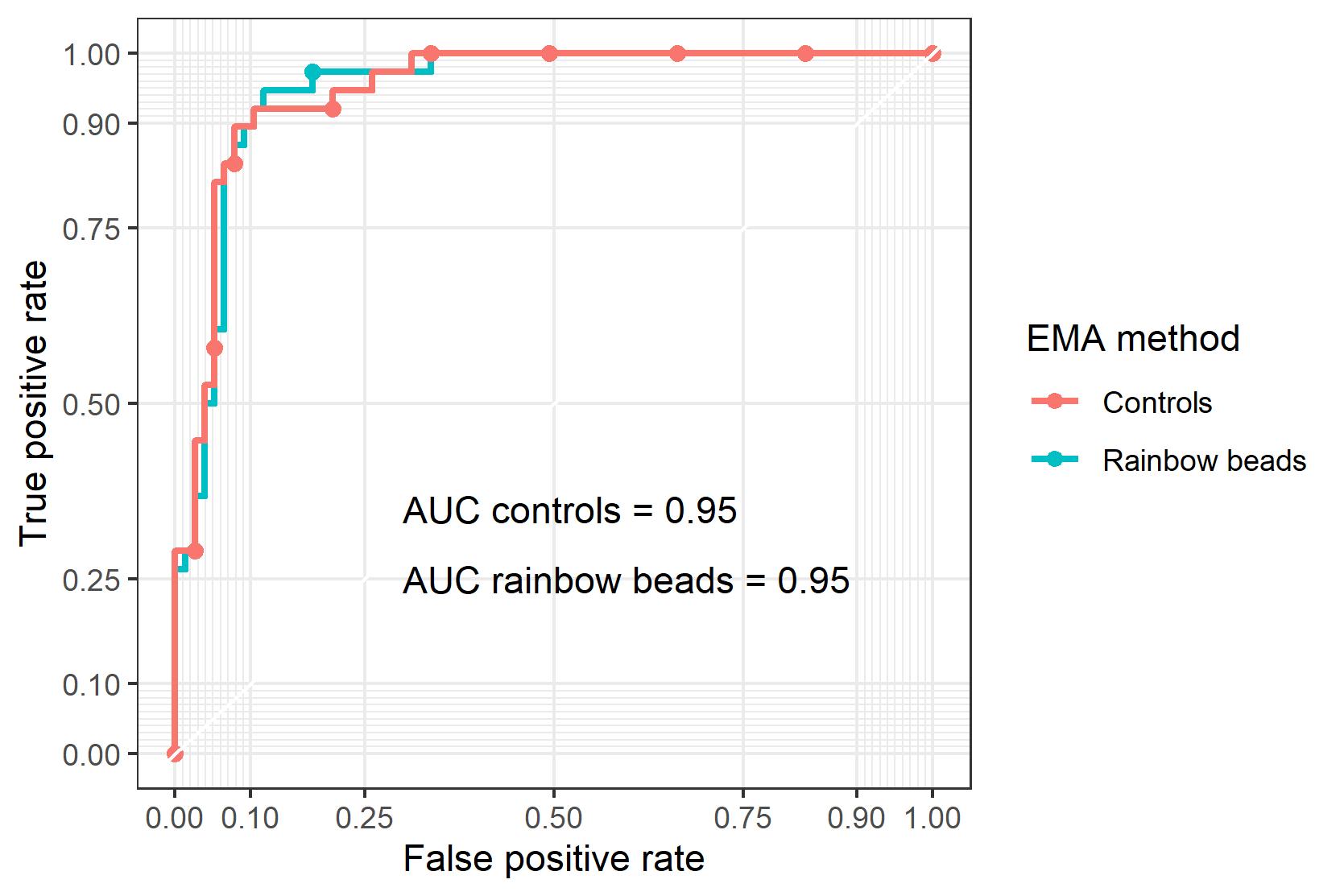

Supplement: FIGURE S2 — Receiver operating characteristic (ROC) curves of EMA binding test either two controls or rainbow beads using osmotic gradient ektacytometry as gold standard (Figure 1B). 87 of the 289 patients had controls with mean fluorescence intensity difference >1000 and were discarded. [file Image_2.JPEG]
